# Childhood Buddy Hangs Up

**DOI:** 10.4103/0973-1229.27601

**Published:** 2006

**Authors:** Ajai Singh

‘R’ really was special. Shouldered the responsibility of a business family at a tender age. Worked over inefficient estate management by elders, saw to it younger siblings were settled, sacrificed youth to bring up business and family. Enjoyed friends, drinks, driving.

We were childhood buddies from the same town.

I had met him a couple of months before the incident. It was after nearly a decade. At a chemist's shop. He was buying medicines. Antidepressants. I asked him what happened. He had tears in his eyes. There was no flicker of the customary light on his face I had known so well. The smile that usually sparkled in his eye as he met an old childhood buddy had vanished.

I could read the distress. I shook hands and told him to meet me in the clinic and we would sort it out. Depression is perfectly treatable, I said. Did you undergo psychotherapy? No, he said. Only drugs, but felt better. Some thoughts bothering you, I asked. Yes. Why not talk to your psychiatrist, I said. He thought for a moment, a long moment. I had never known ‘R’ to take that long to decide. Finally he said he would come and meet me in my clinic.

Suddenly ‘R’ was dead.

Died by hanging.

I wondered what went wrong. Would I have saved him if I had got over my professional reserve and insisted he come for treatment? Was he really taking treatment with someone, or just self-medicating himself? I know he listened to me. If I had phoned him up, or his parents, and told them: nothing doing, I want to see ‘R’ well. Let him come to my clinic. Would that not have given him a chance to survive? Or even if he did commit the act, it would not be for want of trying.

Some days later. As I neared his house while I was going to a neighbour's, I looked up at the forlorn structure. A grim board outside said, “Trespassers will be prosecuted”.

What about the late owner, who prosecuted me since I could not trespass a professional limitation: don't solicit patients.

